# Pulpal sequelae after trauma to anterior teeth among adult Nigerian dental patients

**DOI:** 10.1186/1472-6831-7-11

**Published:** 2007-08-31

**Authors:** Adeleke O Oginni, Comfort A Adekoya-Sofowora

**Affiliations:** 1 Department of Restorative Dentistry, Faculty of Dentistry, College of Health Sciences, Obafemi Awolowo University, Ile-Ife, Nigeria; 2 Department of Child Dental Health, Faculty of Dentistry, College of Health Sciences, Obafemi Awolowo University, Ile-Ife, Nigeria

## Abstract

**Background:**

Epidemiological studies show that about 11.6% to 33.0% of all boys and about 3.6% to 19.3% of all girls suffer dental trauma of varying severity before the age of 12 years. Moderate injuries to the periodontium such as concussion and subluxation are usually associated with relatively minor symptoms and hence may go unnoticed by the patient or the dentist, if consulted. Patients with these kinds of injuries present years after a traumatic accident most of the time with a single discoloured tooth. This study sets out to document the incidence of various posttraumatic sequelae of discoloured anterior teeth among adult Nigerian dental patients.

**Methods:**

One hundred and sixty eight (168) traumatized discoloured anterior teeth in 165 patients were studied. Teeth with root canal treatment were excluded from the study. Partial obliteration was recorded when the pulp chamber or root canal was not discernible or reduced in size on radiographs, total obliteration was recorded when pulp chamber and root canal were not discernible. A retrospective diagnosis of concussion was made from patient's history of trauma to the tooth without abnormal loosening, while subluxation was made from patient's history of trauma to the tooth with abnormal loosening.

**Results:**

Of the 168 traumatized discoloured anterior teeth, 47.6% and 31.6% had partial and total obliteration of the pulp canal spaces respectively, 20.8% had pulpal necrosis. Concussion and subluxation injuries resulted more in obliteration of the pulp canal space, while fracture of the teeth resulted in more pulpal necrosis (p < 0.001). Injuries sustained during the 1^st ^and 2^nd ^decade of life resulted more in obliteration of the pulp canal space, while injuries sustained in the 3^rd ^decade resulted in more pulpal necrosis.

**Conclusion:**

Calcific metamorphosis developed more in teeth with concussion and subluxation injuries. Pulpal necrosis occurred more often in traumatized teeth including fractures.

## Background

Epidemiological studies show that about 11.6% to 33.0% of all boys and about 3.6% to 19.3% of all girls suffer dental trauma of varying severity before the age of 12 years [1-3]. The male: female ratio ranged from 1.3–2.3:1 [1-3]. In Nigeria, the prevalence of traumatized anterior teeth in rural population has been reported to be 6.5% [4] while in the metropolitan population; it is much higher, 14.5% [5]. The number, type and severity of dental injuries differ according to the age of the patient and the cause of the accident. Most of the time, these results in coronal fractures that are easily recognizable by both the patients and their parents, and are also easy to diagnose by the dental practitioner [6]. Moderate injuries to the periodontium such as concussion and subluxation are usually associated with relatively minor symptoms and hence may go unnoticed by the patient or the dentist, if consulted [7]. The maxillary central incisors were the most frequently injured teeth in all studies. While many studies reported the maxillary lateral incisors as the second most frequently injured teeth that of Forsberg and Tedestam [8] reported the mandibular central incisors as the second most frequently injured teeth.

Concussion may be defined as an injury to the tooth supporting structures without abnormal loosening or displacement of the tooth but with marked reaction to percussion. Subluxation is an injury to the tooth supporting structures with abnormal loosening, but without displacement of the tooth. Patients with these kinds of injuries present years after a traumatic accident most of the time with a single discoloured tooth. This discolouration may be the result of obliteration of the pulp canal space, the pulp cavity being filled with dark tertiary dentine resulting in a tooth with less translucent appearance. Analysis by means of scanning and transmission electron microscopy shows that the tissues occluding the pulpal lumen are either dentine like (49%), bone like (19%), or fibrotic (9%) which could not be correlated with explicit clinical diagnoses [9]. This calcific metamorphosis may be recognized clinically as early as 3 months after injury [10]. The pulp calcification and subsequent discolouration increases with time.

Approximately 3.8% to 24% of traumatized teeth develop varying degrees of obliteration. Studies indicate that pulpal necrosis will develop in about 1%–16% of these [10]. While pulpal necrosis only occurs in 3% of teeth subjected to concussion [11]. Following a severe traumatic injury to permanent immature teeth, the growth of calcified tissue in pulp canal space may occasionally occur [12]. Also the pulp may become necrotic leading to the formation of a periapical lesion around a wide-open apex. All these presents various endodontic challenges to the dentist, in cases of symptomatic teeth with partial or complete obliteration of the pulp canal space, root canal treatment may become a difficult or an impossible task respectively [13]. In traumatic teeth with periapical lesion and open apexes, it will be difficult to get a hermetic apical seal with conventional root canal treatment.

The present study sets out to document the incidence of various post traumatic sequelae in discoloured anterior teeth among adult Nigerian patients attending the Dental Hospital of the Obafemi Awolowo University, Ile-Ife, Nigeria.

## Methods

One hundred and sixty eight (168) traumatized discoloured anterior teeth in 165 patients (95 males and 70 females) were studied. Their ages ranged from 20–56 years (mean age ± SD 31.3 ± 8.6 years). These included all patients presenting with traumatized discoloured anterior teeth between August 2003 and July 2005 at the Oral Diagnosis Unit and the Conservative Clinic of the Dental Hospital, Obafemi Awolowo University Ile-Ife, Nigeria. The traumatized discoloured teeth may or may not be the cause of presenting complaint. Discoloured teeth with root canal treatment were excluded from the study, so also were discoloured teeth with no history of reported injury/trauma.

Information extracted from the patients include the history of the discoloured tooth, was there any previous injury/trauma to the tooth? If yes, how long ago was it? How long after the injury/trauma was the discolouration first noticed? Is the discolouration increasing? Has there been any other associated symptom such as pain, swelling, and discharge from the gum around the tooth (sinus tract)? On examination, any fracture or loss of tooth structure, intrusion or extrusion was recorded. Results of sensibility test and radiographic examinations were also recorded. Was there obliteration of the pulp canal space, and/or apical radiolucency? Was the root formation complete or incomplete? Partial obliteration was recorded when the pulp chamber or root canal was not discernible or reduced in size on radiographs, total obliteration was recorded when pulp chamber and root canal were not discernible. A retrospective diagnosis of concussion was made from patient's history of trauma to the tooth without abnormal loosening, while subluxation was made from patient's history of trauma to the tooth with abnormal loosening. The diagnosis of pulpal status was based on a combination of coronal discolouration, sensibility test, clinical symptoms, and radiographic evaluation [6].

Data were subjected to descriptive and statistical analyses using SPSS for windows statistical software package Version 11.0. A significance level p < 0.05 was defined as statistically significant.

## Results

**Figure 1:**
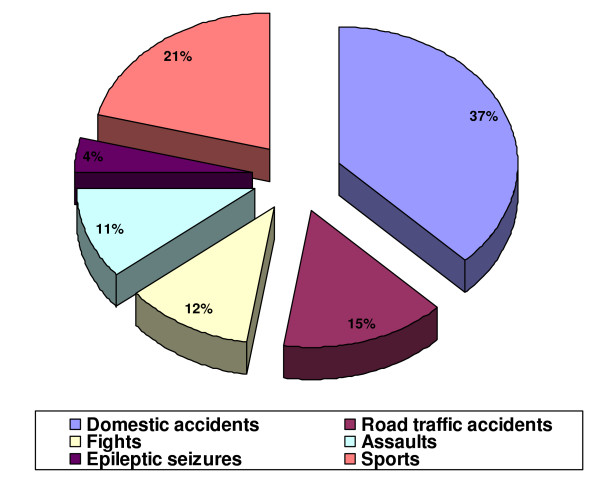
Causes of trauma.

**Figure 2:**
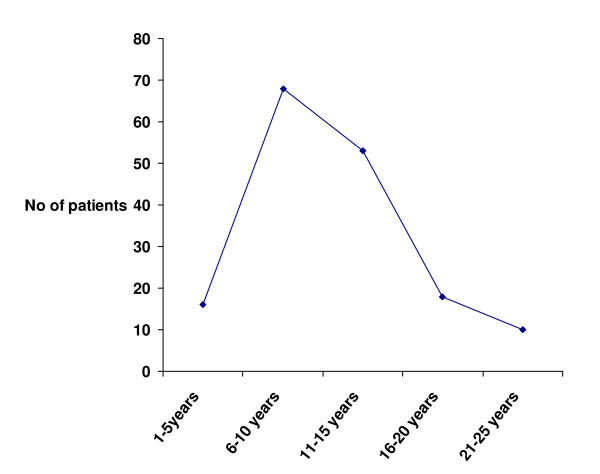
Time lapse between trauma and presentation of discoloured teeth.

A total of 165 patients (95 male, 70 female) presented with 168 traumatized discoloured anterior teeth, with a male: female ratio of 1.36:1. All the discoloured teeth included in this study had histories of some form of traumatic injury leading to fracture of the dental hard tissues in 38(22.6%) of cases, concussion in 53(31.6%) of cases and subluxation in 77(45.8%) of cases. Causes of injuries were domestic accidents (Impact with person, impact with objects, fell or pushed), sports, road traffic accidents (RTA), fights (Physical combat), assault (Abuse), and epileptic seizures (Figure 1). The discolouration resulting from the traumatic injuries were first noticed 4–24 months (mean = 13.2 months and median = 11.0 months) after injury and the discolourations increased with time. The age of the patients at the time of injury ranged from 7 to 30 years (mean age ± SD 14.2 ± 6.1 years). About 60.1% of injuries had occurred by age 12. Figure 2 shows the time lapse between trauma and presentation of discoloured teeth, majority of patients presented 6–10 years after trauma.

Of the 168 traumatized discoloured anterior teeth (167 maxillary incisors; 150 centrals, 17 laterals and 1 mandibular central incisor), 133(79.2%) had obliteration of the pulp canal spaces; partial obliteration in 80(47.6%) of cases, and total obliteration in 53(31.6%) of cases. Thirty-five (20.8%) had necrosis of the pulp out of which 29 had closed apexes and 6 had open apexes (Table 1). Fifty-six, (70.0%) and 26.4% of teeth that had partial and total obliteration of the pulp canal space respectively presented with pain and also showed pathological periapical changes. Teeth with pulp necrosis presented with pain in 51.4%, swelling in 34.4%, and sinus tract in 14.3% of cases. Table 2 shows that concussion and subluxation injuries resulted more in obliteration of the pulp canal space, while fracture of the teeth resulted in more pulpal necrosis. The differences were statistically significant (p < 0.001). Partial obliteration of the pulp canal space occurred more frequently from all the injury types than total obliteration, the differences were not statistically significant (p > 0.05), Table 2. In 72(42.9%) of cases, the injury to the teeth was sustained during the first decade of life, while in 32.7% and 24.4% of cases, the injury occurred during the 2^nd ^and 3^rd ^decade of life respectively. Obliteration of the pulp canal space was more frequent in teeth that were traumatized during the 1^st ^and 2^nd ^decade of life, while pulpal necrosis was more frequent in teeth traumatized during the 3^rd ^decade of life. The differences were statistically significant (p < 0.001), Table 3. Pulpal necrosis occurred more frequently in fractured teeth. Fracture, secondary to road traffic accident (RTA) resulted to pulpal necrosis more in teeth traumatized during the 3^rd ^decade of life.

**Table 1:** Incidence of post traumatic sequelae

Post traumatic sequelae	No (%)
Partial obliteration	80 (47.6)
Total obliteration	53 (31.6)
Pulp necrosis	35 (20.8)

Total	168 (100.0)

**Table 2:** Injury type and post traumatic sequelae

Injury type	^A^Partial obliteration No (%)	^B^Total obliteration No (%)	^C^Pulpal necrosis No (%)
Fracture (n = 38)	8 (21.0)	6 (15.8)	24 (63.2)
Concussion (n = 53)	28 (52.8)	20 (37.7)	5 (9.4)
Subluxation (n = 77)	44 (57.1)	27 (35.1)	6 (7.8)

(A+B)vsC: χ^2 ^= 53.4, df = 2, p < 0.001; AvsB: χ^2 ^= 0.22, df = 2, p = 0.9

**Table 3:** Age at time of injury and post traumatic sequelae

Age group (yrs)	^A^Partial obliteration No (%)	^B^Total obliteration No (%)	^C^Pulp necrosis No (%)	Total No (%)
1 – 10	38 (52.8)	29 (40.3)	5 (6.9)	72 (100)
11 – 20	29 (52.8)	17 (30.9)	9 (16.3)	55 (100)
21 – 30	13 (31.7)	7 (17.1)	21 (51.2)	41 (100)

(A+B)vsC: χ^2 ^= 31.57, df = 2, p < 0.001; AvsB: χ^2 ^= 1.07, df = 2, p = 0.59

## Discussion

To determine the frequency of calcific metamorphosis in traumatized teeth, it would have been better to follow-up traumatized teeth for a long period of time. However, from our experience, response to recall and follow-up visit is very poor. Therefore, it was decided to look into the incidence of calcific metamorphosis and pulpal necrosis in patients presenting with discoloured anterior teeth secondary to traumatic injuries. The study was carried out in Southwestern Nigeria; hence the population studied may not be representative of the total Nigerian population.

Most international surveys reported that males experienced significantly more dental trauma to the permanent dentition than females [14,15]. In this study, we got a male: female ratio of 1.36:1, this falls within the usually quoted range of 1.3–2.3:1 [1-3]. However, a lower ratio of 0.9:1.0 has been reported for children less than seven years old [16]. Domestic accidents accounted for most of the injuries in the present study (37.0%), this is in agreement with earlier studies [16,17] that reported accidents at home and school to account for most injuries to the permanent dentition.

In the discoloured traumatized anterior teeth presented in this study, subluxations were the most frequent type of injury (45.8%), followed by concussions (31.6%) and fractures (22.6%). These were contrary to the findings of Petti et. al. [18] in which fractures (enamel, 67%; enamel-dentine, 19.3%) were the most frequent type of injury followed by concussions (8.3%). Also Rocha and Cardoso [19] reported fractures (51.4%) to be more frequent than luxation (48.6%). The differences are to be expected since the present study dealt with discoloured teeth secondary to trauma and not a survey of all the traumatized anterior teeth. It may be that patients who sustained severe injury to their teeth resulting in serious fractures had earlier sought treatment, hence the low frequency of fractures in this study. Because of the difficulty in determining the pulpal sequelae in traumatized teeth that have already been treated, they were excluded from the study. Also it is widely accepted that moderate injuries such as concussions and subluxations most of the time go unnoticed. Patients with such injuries usually presents later with discoloured teeth.

The reactions of the dental pulp to traumatic injuries can be extremely varied. They ranged from almost immediate pulp death to long-term slow pulp canal obliterations [20]. In the sequelae of calcific degeneration, the clinical crown frequently becomes discoloured. In this study, obliteration of the pulp canal space was more frequent in concussion and subluxation injuries, while pulpal necrosis was more frequent in fractures. The differences were statistically significant p < 0.001. However, the differences in the frequency of partial or total obliteration of the pulp canal space were not statistically significant (p > 0.05) in relation to the injury type. In the present study, pulpal necrosis occurred in 9.4% of teeth subjected to concussions. This is much higher than the 3.0% reported by Andreasen and Vestergaard Pedersen [11]. The authors could not proffer any reason for this. Injuries sustained during the 1^st ^and 2^nd ^decade of life resulted more in obliteration of the pulp canal space, while injuries sustained in the 3^rd ^decade resulted in more pulpal necrosis. The differences were statistically significant (p < 0.001). It was observed that road traffic accident (RTA) was the major cause of injuries in the 3^rd ^decade of life leading to enamel-dentine fractures.

Although prophylactic endodontic treatment in teeth displaying pulp canal obliteration on a routine basis does not seem justified, it has been reported that the incidence of pulpal necrosis increases over the course of time [21]. In this study, the majority (70.0%) of teeth with partial obliteration of the pulp canal space presented with pain and showed pathologic periapical changes, which may have resulted from pulpal necrosis. However, this runs contrary to the findings of Jacobsen and Kerekes [22] who reported normal periapical conditions in all teeth with partial obliteration. Only 26.4% of teeth with total obliteration presented with pain and showed pathologic periapical changes. This is in partial agreement with the findings of Jacobsen and Kerekes [22] in which 21.0% of teeth with total obliteration developed pathologic periapical changes. From these, teeth with partial obliteration of the pulp canal space are more likely to be symptomatic than those with total obliteration. Although, an earlier study had suggested that increase in the amount of calcification might lead to partial or complete radiographic but not microscopic obliteration of the pulp chamber and root canals [23].

## Conclusion

Calcific metamorphosis developed more in teeth with concussion and subluxation injuries. Pulpal necrosis occurred more often in traumatized teeth including fractures.

## Competing interests

The author(s) declare that they have no competing interests.

## Authors' contributions

AOO conceived of the study, participated in the design and collection of data, carried out examination of patients, performed the statistics, and participated in the initial draft and final write up of the manuscript. CAA participated in the design and collection of data, participated in the initial draft and final write up of the manuscript.

All authors read and approved the final manuscript.

## Pre-publication history

The pre-publication history for this paper can be accessed here:



## References

[B1] Clarkson BH, Longhurst P, Sheiham A (1973). The prevalence of injured anterior teeth in English school children and adults. J Dent Child.

[B2] Jarvinen S (1979). Fractured and avulsed permanent incisors in Finnish children. A retrospective study. Acta Odontol Scand.

[B3] Baghdady VS, Ghose LJ, Enke H (1981). Traumatized anterior teeth in Iraqi and Sudanese children-A comparative study. J Dent Res.

[B4] Otuyemi OD, Sofowora CA (1991). Traumatic anterior dental injuries in selected rural Primary school children in Ile-Ife, Nigeria. Nig Dent J.

[B5] Akpata ES (1969). Traumatised anterior teeth in Lagos school children. Nig Med J.

[B6] Andreasen JO, Andreasen FM (1994). Textbook and Color Atlas of Traumatic Injuries to the teeth.

[B7] Ebeleseder KA, Glockner K (1999). Diagnostik des dentalen Traumas-Erstuntersuchung und Verletzungsarten. Endodontie.

[B8] Forsberg CM, Tedestam G (1990). Traumatic injuries to teeth in Swedish children living in an urban area. Swed Dent J.

[B9] Robertson A, Lundgren T, Andreasen JO, Dietz W, Hoyer I, Noren JG (1997). Pulp calcifications in traumatized primary incisors. A morphological and inductive analysis study. Eur J Oral Sci.

[B10] Amir FA, Gutmann JL, Witherspoon DE (2001). Calcific metamorphosis: a challenge in endodontic diagnosis and treatment. Quintessence int.

[B11] Andreasen FM, Vestergaard Pedersen B (1985). Prognosis of luxated permanent teeth-Development of pulp necrosis. Endod Dent Traumatol.

[B12] Heling I, Slutzky-Goldberg I, Lustmann J, Ehrlich Y, Becker A (2000). Bone-like tissue growth in the root canal of immature permanent teeth after traumatic injuries. Endod Dent Traumatol.

[B13] Ngeow WC, Thong YL (1998). Gaining access through a calcified pulp chamber: a clinical challenge. Int Endod J.

[B14] Zerman N, Cavalleri G (1993). Traumatic injuries to permanent incisors. Endod Dent Traumatol.

[B15] Kaba AS, Marechaux SC (1989). A fourteen-year follow-up study of traumatic injuries to the permanent dentition. J Dent Child.

[B16] Onetto JE, Flores MT, Garbarino ML (1994). Dental trauma in children and adolescents in Valparaiso, Chile. Endod Dent Traumatol.

[B17] Caliskan MK, Turkun M (1995). Clinical investigation of traumatic injuries of permanent incisors in Izmir, Turkey. Endod Dent Traumatol.

[B18] Petti S, Tarsitani G, Arcadi P, Tomassini E, Romagnoli L (1996). The prevalence of anterior tooth trauma in children 6 to 11 years old. Minerva Stomatol.

[B19] Rocha MJ, Cardoso M (2001). Traumatized permanent teeth in Brazilian children at the Federal University of Santa Catarina, Brazil. Dent Traumatol.

[B20] Feiglin B (1996). Dental pulp response to traumatic injuries – a retrospective analysis with case reports. Endod Dent Traumatol.

[B21] Robertson A, Andreasen FM, Bergenholtz G, Andreasen JO, Noren JG (1996). Incidence of pulp necrosis subsequent to pulp canal obliteration from trauma of permanent incisors. J Endod.

[B22] Jacobsen I, Kerekes K (1977). Long-term prognosis of traumatized permanent anterior teeth showing calcifying processes in the pulp cavity. Scand J Dent Res.

[B23] Piatteli A (1992). Generalized "complete" calcific degeneration or pulp obliteration. Endod Dent Traumatol.

